# ﻿Lectotypification of *Pterocaryaserrata* C.K.Schneider (Juglandaceae), the name of a forgotten species of wingnuts

**DOI:** 10.3897/phytokeys.260.158522

**Published:** 2025-08-01

**Authors:** Susanne S. Renner, Michael S. Dosmann

**Affiliations:** 1 Department of Biology, Washington University, Saint Louis, MO 63130, USA Washington University Saint Louis United States of America; 2 Arnold Arboretum of Harvard University, 125 Arborway, Jamaica Plain, Boston, Massachusetts 02130, USA Arnold Arboretum of Harvard University Boston United States of America

**Keywords:** Camillo K. Schneider, E.H. Wilson, Juglandaceae

## Abstract

The Juglandaceae species *Pterocaryaserrata* C.K.Schneid. was first described in 1912, but its assumed holotype, a flowering E. H. Wilson collection in the Vienna herbarium, was destroyed during World War II. Schneider described *P.serrata* as ‘fruits unknown’ because he thought that a fruiting specimen, collected under the same number, was a mixed collection. Pre-1958, citation of a single collection does not automatically constitute a type, but we here accept the Vienna material as the lost holotype and select the A duplicate with the most precise location information as the lectotype, with isolectotypes in at least six other herbaria. Additionally, we designate a fruiting specimen as the epitype.

Based on a flowering collection from western Hubei, China, made in June 1900 by the British collector E.H. Wilson (1876–1930), the German landscape architect and botanist C.K. Schneider (1876–1951) described *Pterocaryaserrata*, a wingnut species with relatively large leaves. He compared it to *P.stenoptera* C.DC, one of the six then-known species of wingnuts (*ptero* meaning wing). Despite the availability of multiple duplicates of W*ilson 901 June 1900* in European and North American herbaria (below) and despite Schneider’s widely recognized taxonomic expertise --he published numerous taxa in diverse families--, *P.serrata* fell into oblivion. A factor contributing to this may have been that Schneider described it as ‘fruits unknown’ although he assumed that it had *Pterocarya*-like winged fruits. This is clear from his statement in the protologue that “This species is deposited in the Herbarium Hofmeister [in] Vienna as Wilson No. 901, VI 1900, W-Hupei [Hubei] in flower and mixed with a young infructescence that probably belongs to No. 546, *Pt.paliurus* [Batalin]” (Schneider 1912, translated from the German by SSR). The fruiting collection *Wilson 546* was made in September 1900 and is preserved at K (barcode K004053898). Without DNA from this specimen, it is not possible for us to decide whether it represents *P.paliurus* or *P.serrata*.

While Schneider in 1912 only knew the *Wilson 901* herbarium material, which he probably received from Veitch, Wilson himself seems to have been aware that the species he was collecting has disk-like, not pointy-winged fruits, and he considered all his collections as representing *P.paliurus*. This is apparent from the treatment in “*PlantaeWilsonianae*” (Sargent 1917, p. 182), which states under *P.paliurus* “Western Hupeh: north and south of Ichang, woods, alt. 1000–1600 m., June and October 1907 (No. 452; tree 13–20 m. tall, 1.5–2.5 m. girth); same locality, June 1900 (Veitch Exped. No. 901; flowers); Kui-chou, August 1900 (Veitch Exped. No. 901; fruit).” Wilson used different numbering systems while working for Veitch and after moving to the Arnold Arboretum in late 1906 (Howard 1980), and the locations given in Sargent (1917) for the flowering and fruiting *901* collections are more precise than the label information on specimens in A, E, K, LE, P, and NY, which are mostly labeled as ‘W Hupeh’ or on the lectotype selected here (below) as ‘China, Chienshi.’ The latter precise location in Western Hubei agrees with Wilson’s itinerary of June 1900 (Howard 1980: p. 135 Chienshi Hsien).

A species described with ‘fruits unknown’ --but assumed to have *Pterocarya*-type pointy-winged fruits—combined with the existence of duplicates of *Wilson 901* showing disk-winged fruits that looked like those of *P.paliurus*, understandably resulted in *P.serrata* being considered a synonym of *P.paliurus*, although we are unaware of a formal synonymization of the name. Iljinskaya (1953) recognized *P.serrata*, but a taxonomic treatment of *Pterocarya* by Manning (1975) does not mention the name and the English-language Juglandaceae treatment for the “*Flora of China*” (Lu et al. 1999) does not either. Duplicates of Wilson’s flowering June 1900 and fruiting August 1900 collections in HUH and Paris were not annotated as *P.serrata* prior to this study, and the NY and Paris duplicates were found among undetermined Juglandaceae.

Recent field work and genomic data reveal that Schneider’s *P.serrata* probably should be considered a reproductively isolated (‘biological’) species that is close to *P.paliurus* but differs in leaf and fruit size, chromosome number, and ecology (Song et al., in review). This discovery led us to study Wilson’s material of *P.serrata* and to lectotypify the name. Given that the name was published pre-1958, citation of a single collection in the protologue does not automatically constitute a type (Turland et al. 2018). It seems likely, however, that the flowering duplicate of *Wilson 901* collected in June 1900 and cited by Schneider (1912) can be considered the sole basis for the description and thus the holotype, despite Schneider’s mention of the loose infructescence that he considered to belong to *P.paliurus*. Unfortunately, most Juglandaceae material, including Schneider’s *P.serrata*, was destroyed in a fire in July 1945 (Rubinstein and Dosmann 2020; T. Schuster, pers. comm. to SSR on 23 April 2025).

Camillo Karl Schneider lived and worked in Berlin and Vienna. In 1913, supported by the Austro-Hungarian Dendrological Society, he went to China to collect plants and seeds, and on the way back, he visited the Arnold Arboretum in Boston, where he worked with Wilson, Charles S. Sargent (1841–1927) and Alfred Rehder (1863–1949). Together, these workers dealt with the thousands of Chinese collections made by Wilson, resulting in the three-volume “*PlantaeWilsonianae: An Enumeration of the Woody Plants Collected in Western China for the Arnold Arboretum of Harvard University During the Years 1907, 1908, and 1910 by E. H. Wilson*” (Sargent 1913, 1916, 1917). The collaboration between Schneider and Wilson from 1913/1914 to 1919 may have influenced these workers’ understanding of *P.serrata*, but if so, this happened after Schneider’s (1912) description of the species. A bit surprisingly, the name is not mentioned in Rehder and Wilson’s treatment of the Juglandaceae in “*PlantaeWilsonianae*” (Sargent 1917).

Enquiries about the presence of duplicates of W*ilson 901 June 1900* in the herbaria A, B, BM, E, GH, K, KYO, LE, NY, P, and W, showed that A, E, K, LE, NY, and P harbor this collection. Since the first set of the Wilson material is at HUH, we here select one of Harvard’s two sheets of W*ilson 901 June 1900* as the lectotype (Fig. 1), namely the one labeled as from Chienshi [today Chien-shih-hsien, alternative name Chien-Shih]. Chien-shih-hsien is located at 30°37'1"N, 109°37'58"E.

**Figure 1.**
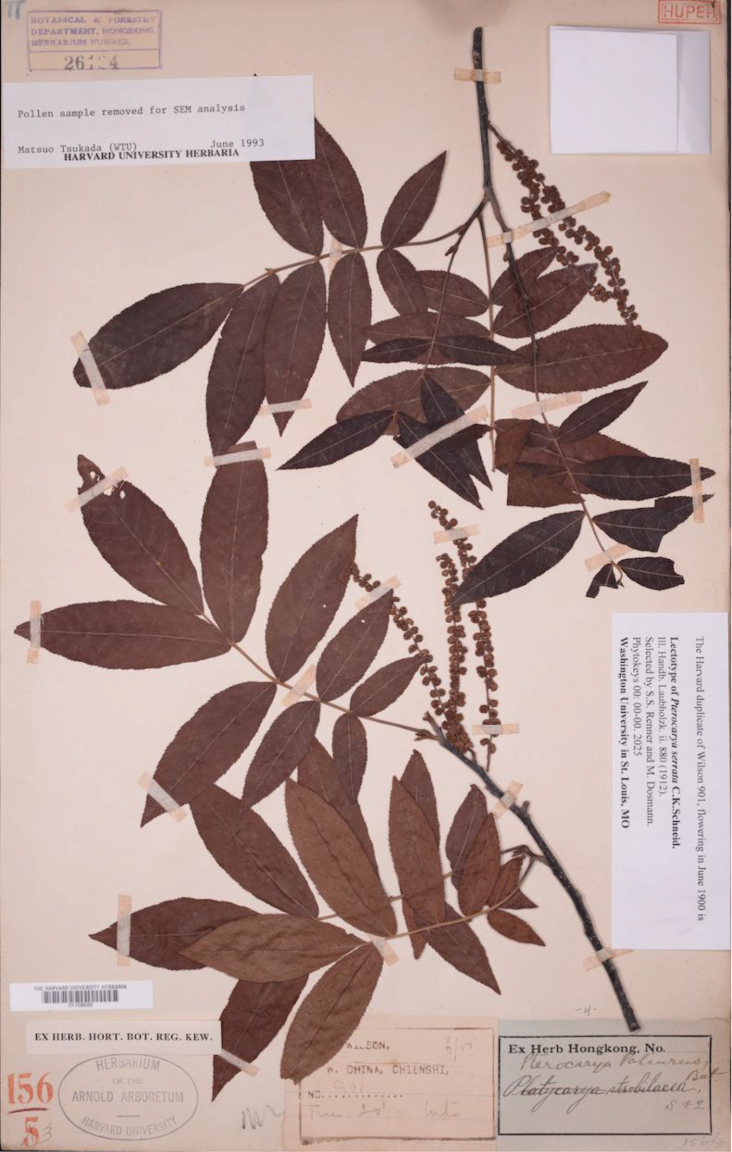
Wilson 901, collected at Chien-Shih (Chien-shih-hsien) in June 1900, the lectotype of *Pterocaryaserrata* C.K.Schneid. Credit line: The Herbarium of the Arnold Arboretum of Harvard University.

## ﻿Typification

### ﻿ Pterocarya serrata

Taxon classificationAnimaliaFagalesJuglandaceae

C.K.Schneid., Ill. Handb. Laubholzk. 2: 880 (1912).

7382AD67-95EC-5079-9F90-DE6A4E917E98

#### Type.

China: • Western Hubei: Chien-shih-hsien, flowering in June 1900, E. H. Wilson 901 p.p., lectotype designated here: A (barcode A01109592), Isolectotypes: A (barcode A01109593); E (barcode E00275520), annotated as an isotype of *P.serrata* by W. E. Manning in July 1963; K (barcode K004053893), comprising a flowering sprig and two loose fruits), K (barcode K004053899), annotated as *P.paliurus* by W. E. Manning in Jul. 1962; LE (barcode LE 01070443), comprising two flowering sprigs collected in June and two loose fruits; NY (barcode NY00255120), annotated as an isotype of *P.serrata* by W. E. Manning in Mar. 1971, NY (barcode NY4358432), annotated as *P.paliurus* by W. E. Manning in Mar. 1971, P barcode (P06842578), annotated as *P.paliurus* by W. E. Manning in Oct. 1965.

***Epitype***: • Fruiting in August 1901, E. H. Wilson 901 p.p: designated here: A (barcode A 01109518). Other Wilson 901 collections from August 1900 are preserved at E (barcode E00275519), K (barcode K004053892), P (barcode P06842583), and NY (barcode 04358771), and K has another undated flowering Wilson 901 sheet (barcode K004053894).

Annotation labels for all Wilson 901 collections from both June and August 1900 have been sent to all these herbaria.

The disk-like fruits of *P.paliurus* led the Russian paleobotanist Irina Alekseevna Iljinskaya (1921–2011) to move this species into a separate genus, *Cyclocaryapaliurus* (Batal.) Iljinsk. (Iljinskaya 1953, 1990, 1994), an up-ranking not accepted by Manning (1975, 1978), but followed by Stone (1993), Manos et al. (2007), and Manchester et al. (2009). If the genus *Cyclocarya* is accepted, then the name *P.serrata* should be transferred to that genus.

## Supplementary Material

XML Treatment for Pterocarya serrata

## ﻿Additional information

### Use of AI

No use of AI was reported.

### Author ORCIDs

Susanne S. Renner https://orcid.org/0000-0003-3704-0703

### Data availability

All of the data that support the findings of this study are available in the main text.
